# Dexamethasone-Loaded biodegradable magnetic microparticles for treatment of CFA-induced chronic pain in rats

**DOI:** 10.1080/15685551.2022.2068743

**Published:** 2022-04-28

**Authors:** Jin Xin, Zheng Jichun, Sun Yonghai

**Affiliations:** a Anesthesiology Department of the Chinese PLA Air Force Medical Center, Beijing, Hebei, China; b Pathology Department of the Chinese PLA Air Force Medical Center, Beijing, Hebei, China; c Department of Comprehensive Treatment, The Second Medical Center of the Chinese PLA General Hospital, Beijing, Hebei, China

**Keywords:** chronic inflammatory pain, PLGA microparticles, dexamethasone, drug release, biocompatibility, magnetic therapy, local injection

## Abstract

Traditional drug solutions or suspensions, have been shown to treat pain in complete Freund’s adjuvant (CFA)-induced chronic inflammatory pain in rats, with or without combination with magnetic therapy. In this study, we aimed to prepare, characterize, and evaluate the therapeutic effects of microparticles containing dexamethasone for local administration and treatment of chronic inflammatory pain. The results showed the following; a) Preparation and characterization: two ratios of poly(lactic-co-glycolic acid) (PLGA)/poly(lactic acid) (PLA) were used. The prepared batches were similar in size and magnetic responsiveness. The microparticle size distribution assessed via electron microscopy suggested a homogeneous distribution and absence of aggregates. Dexamethasone release profiles (microparticles synthesized with a feed ratio of 1:4) showed a sustained release in vitro and good biocompatibility with tissues. b) Therapeutic effect: the treatment effect of dexamethasone-PLGA magnetic microspheres + magnetic therapy was substantially better than that observed for other groups on day 4, as monitored by appearance, mechanical pain threshold, and histological analysis. This type of carrier could be a suitable magnetically retainable local drug delivery system for treating chronic pain.

## 1. Introduction

Chronic pain affects millions of patients adversely, affecting their quality of life.Many of these patients suffer for years undergoing mental, physical, and financial burdens, including insomnia and depression, even loss of jobs and physical strength [1,2]. These patients require frequent treatments, thus getting deeply troubled by the cost and frequency of chronic pain treatment schedules [2]. Local administration of corticosteroids is an effective method for treating chronic pain. [3,4]Thus, novel corticosteroid-containing poly(lactic-co-glycolic acid) (PLGA)/ poly lactic acid (PLA) microspheres can be used for localized administration on lesions, hence prolonging the effect of drugs due to sustained release [5,6]. Their application not only reduces the frequency of treatment but also decreases the cost. Long-term side effects such as osteoporosis and hormone dependence are also reduced. There have been a few studies on the treatment of chronic pain by local injection of microspheres loaded with local anesthetics [7,8]. As chronic pain is always caused by chronic inflammation, corticosteroids, particularly dexamethasone, are one of the commonly prescribed treatments for chronic inflammation. In addition, some studies have reported the synthesis of dexamethasone-PLGA microspheres. These studies have demonstrated the efficacy of intra-articular dexamethasone-PLGA microsphere administration in treating arthritis [9–12]. Despite their undeniable advantage, dexamethasone-loaded PLGA microspheres are rapidly cleared from tissues through macrophage uptake or lymphatic drainage. This limitation can be overcome by using microparticles rendered magnetizable by the encapsulation of superparamagnetic iron oxide nanoparticles, and managing their localization through an external magnetic field.

Local administration of dexamethasone, a potent corticosteroid, has been shown to effectively control chronic inflammation [10]. However, delayed tissue reactions always occur after the drug is exhausted. Dexamethasone-loaded PLGA microsphere/ polyvinyl alcohol (PVA) hydrogel composites were therefore used to control the foreign body reaction for 3 months [9,11,13,14]. Dexamethasone-PLGA microspheres exhibit a triphasic drug release profile: a burst release phase, a lag phase, and a secondary release phase. As the course of chronic inflammatory pain is always a long-term process, a sustained release of dexamethasone is more suitable for such patients [10,12–16]. In this study, complete Freund’s adjuvant (CFA) was injected into the plantar to induce chronic nociception and simulate chronic inflammatory pain in rats. The pathological features, in the CFA-induced rat pain model, were similar to those observed in chronic inflammatory pain and characterized by a granulomatous appearance.

In the present study, we prepared dexamethasone-loaded PLGA/PLA magnetic microspheres. Magnetic particles can be injected locally into the target area in rats, and once localized, they can be excited by an external magnetic field. However, whether it could be administered intravenously or not, needs to be further tested. Notably, magnetic targeting can only be used to localize particles larger than 1.0 µm at the desired site [11–13]. The particles we prepared were much larger than the standard, as it is not practical to locate nanometer-sized particles under the influence of a magnetic field. Although large, the particles remain suspended in solution by motion, and if the system flow is subsistent, they can be transported with flow. The magnetic microspheres move in a magnetic field, which leads to drug accumulation at the affected site [17]. Additionally, particle movement at the affected place results in a physical therapy-like effect [18]. The behavior of particles in the magnetic field is thus beneficial for the local treatment of inflammatory pain; therefore, microspheres were preferred. In this study, we describe the fabrication, in vitro drug release, stability evaluation, biocompatibility, and therapeutic effect of microspheres in a CFA-induced chronic inflammatory rat model [19].

## 2. Experiments and Methods

### 2.1. Drug experiments

#### 2.1.1. Materials

PLGA (50:50) was obtained from Shandong Academy of Pharmaceutical Sciences (China). Dexamethasone 21-acetate was purchased from Sigma Aldrich (Switzerland). PVA was purchased from Kuraray (Japan). Nanometer iron powder was obtained from Sichuan Jinsha Nanotechnology Co. Ltd. (China).

#### 2.1.2. Synthesis of nanoparticles

**Figure 1.**
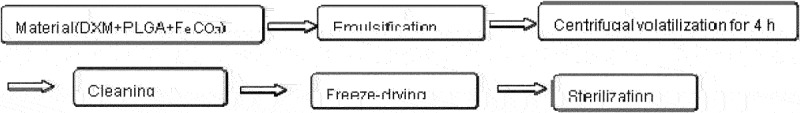
Preparation process of drug-loaded microspheres.

The microspheres were prepared using the solvent volatilization method (S/O/W), with a PLGA/Fe_3_O_4_ ratio of 4:1. The specific mass of the different components was calculated according to different feed ratios (dexamethasone mass/total mass of material used). The rotational speed was 1800 r/min and volatilization lasted for 4 h until dichloromethane was completely evaporated. After solidification, the microspheres were filtered through a 0.20 µm filter membrane under vacuum and negative pressure. The microspheres were collected and lyophilized after washing with deionized water and stored at 4 °C. (Figure 1)

In the study, microspheres were categorized according to different feed ratios (DXM mass/total mass of materials used) of 1:3, 1:4, and 1:5 (Table 1). The mass ratio of PLGA/iron powder was 4:1. For analyzing particle size, the particle size of at least 500 microspheres was determined, and the arithmetic mean diameter was calculated.

**Table 1.** Formula of microspheres with different feed ratios

Feed ratio	DXM (mg)	(PLGA + Fe_3_O_4_) (mg)	Total mass (mg)
1:3	100	200(150 + 50)	300
1:4	100	300(240 + 60)	400
1:5	100	400(320 + 80)	500

Ps: DCM: dichloromethane; PVA: Polyvinyl Alcohol

Ps: PLGA/Fe_3_O_4_ = 4/1; feed ratio = Dexamethasone (DXM) mass/total mass of material used.

#### 2.1.3. Morphologic and size studies

Dexamethasone-PLGA magnetic microspheres were coated on a slide, dispersed with an appropriate amount of double steam water, observed, and imaged at a magnification of 400× using a scanning electron microscope.

(1) $$Span=\;\frac{D90-D10}{D50}$$

Ps:D90, D50, and D10, respectively, indicate the numbers of microspheres with diameter less than 90%, 50%, and 10% of all the microspheres.

#### 2.1.4. Assessment of microspheres

Assessment of microspheres was based on the following aspects: sum value (S) of span (S1), yield (S2), encapsulation rate (S3), and drug loading (S4) of microspheres, was evaluated, the formula is $\begin{array}{rl} & S=-S1+S2+S3+S4 \end{array}$. [9,20,21]

The calculation method for span (S1) is shown above in Equation (1).

(2) $$microsphere\;yield\;(S2)=\frac{wt\;of\;all\;ingredients\;entrapped}{wt\;of\;all\;ingredients\;used}\times 100\%$$

(3) $$DXM\;encapsulationrate\;\left(S3\right)=\frac{wt\;of\;entrapped\;DXM}{wt\;of\;DXM\;used}\times 100\%$$

(4) $$DXM\;loading\;(S4)=\frac{wt\;of\;DXM\;entrapped}{wt\;of\;microparticles\;used\;for\;dosing}\times 100\%$$

Ps. DXM: Dexamethasone

***Stability of microspheres***: Different batches of microspheres were placed in a refrigerator at 4–8 °C for 3 months, after which their drug loading, particle size, and other physical properties were measured.

#### 2.1.5. Magnetic responsiveness studies

Dexamethasone-PLGA magnetic microspheres were added to double-steamed water. The distribution and motion of the microspheres were observed under a magnetic field of 4000 GS using a microscope (magnification of 400×). After drying, the adsorbed and unadsorbed microspheres were weighed, and the adsorption rate was calculated using the formula.

(5) $$\;\;\;\;\;\;\;\;\;\;\;\;\;\;\;\;\;\;\;\;\;\;\;\;\;\;\;\;\;\;\;\;\;\;\;\;\;\;\;\;\;\;\;\;\;\;\;\;\;\;\;\;\;\;\;\;\;\;\;\;\;\;\;\;\;\;\;\;\;\;\;\;\;\;\;\;\;\;\;\;\;\;\;\;\;\;\;\;\;\;\;\;\;\;\;magneticadsorptionrate\text{\textbackslash{}break}=\frac{adsorbedmicrospheres}{adsorbedmicrospheres+unadsorbedmicrospheres}\times 100\%$$

#### 2.1.6. Determination of dexamethasone Content

*Chromatography*: Chromatographic column: Hypersil C18 (150 × 4.6 mm, 5 µm); mobile phase: acetonitrile: phosphate buffer (25 mmol L^−1^, pH = 3.0) (45:55); flow rate, 1.0 mL min^−1^; detection wavelength, 240 nm; column temperature, 30 °C.

### 2.2. Animal experiments

#### 2.2.1. Experimental animals

60 adult male Sprague-Dawley rats weighting 220–250 g were purchased from the Animal Center of PLA General Hospital (Beijing, China). Four or five rats were housed in a large cage. Feeding environment of the animals alternated between day and night for 12 h in cages with free access to water and food pellets. Animal housing room was kept free of noise and the temperature and humidity were maintained at 18–22 °C and 40–60% respectively. Food and water should not be ingested for 12 hours before surgery. The rats were tested after 7 days of adaptive feeding. The experimental protocol was approved by the Ethics Committee of the PLA General Hospital.

#### 2.2.2. Anesthesia and treatment methods

Grouping: A total of 36 rats were randomly selected and divided into six groups (n = 6 per group) based on the different treatments and whether they were given combined magnetic therapy (Table 2). The left column of the table shows the positive control group and the single drug group, whereas the right side shows the magnetic therapy group and the combined magnetic therapy group (Table 2). The specific treatments are detailed in the treatment section.

**Table 2.** Animal experimental group

Dexamethasone treatment group	Combined magnetic therapy group
Positive control group (PC, Inflammation model)	Only magnetic therapy group (OMT)
Microspheres dexamethasone group (MD)	MD + MT
Phosphate dexamethasone group (PD)	PD + MT

Anesthesia: After the rats were transferred into the inhalation anesthesia box. Rats were inhaled with 3%-5% sevoflurane (Heng Swee, China) for 5–10 min with 2 L /min oxygen. Until the rats were under moderate anesthesia, the pain disappeared, the righting reflex disappeared, the skeletal muscle relaxed, and the breathing was stable.

Preparation of dexamethasone microsphere suspension: Weigh 100 µg PVA and 10 mL water for injection, mix them, and dissolve them in a water bath at 60 °C for 30 min. (Table 3)

**Table 3.** Suspension and reference formula (mg/mL)

DXM dosage forms	Formula	Note
Microsphere suspension	Microsphere 10 mg + PVA 100 µg + NS 10 mL	0.3 ml = 0.1 mg DXM
Sodium phosphate solution*	DXM (5 mg/ mL)0.2 ml + NS 10 mL	0.1 mL = 0.1 mg DXM

*DXM: Dexamethasone sodium phosphate solution was obtained from RongSheng Co, Ltd, China

Treatment: 0.3 mL of dexamethasone microsphere suspension (approximately 0.1 mg dexamethasone) was extracted with a 1 mL syringe, and the anesthetized rats were placed on the operating table, and subcutaneously injected into the left posterior sole of the rats. After injection, the rats were taken from other cages and placed separately.

Suspension and reference are prepared as follows (Table 3)

#### 2.2.3. Biocompatibility experiments

*In vitro Hemolysis Test*: Acorrding to the methods of supplimentary infomation to preparation of test solution,red blood cell suspension(A),Negative control (B) and Positive control (C).

The above A + B + C samples were thoroughly mixed and incubated in a thermostatic water bath oscillator and maintained at 36–37 °C, for 3 h. The samples were centrifuged at 3000 r /min for 5 min, and the supernatant was removed and left to stand at room temperature for 30 min. The hemolysis rate (HL%) was calculated based on the absorbance value **[**22**]** obtained by spectral scanning at 540 nm. The light absorption values were determined using the negative control as blank standard. Absorbance of negative control was ≤ 0.03, absorbance range of positive control was 0.8 ± 0.3. A Hemolysis rate (%) < 5%, was an indicative that the tested sample had no hemolysis **[**23**]**.

*Blood Compatibility Test In vivo*: Three SD rats were weighed. The mother liquor of the above sample was injected into the rats through the tail vein. Blood (0.5 mL) was collected at 0.5 h, 6 h, 12 h, and 24 h before and after injection, and placed in anticoagulant tubes for routine blood examination.

*Histocompatibility Test*: Rats were anesthetized by inhaling sevoflurane, and hair from the left back was shaved off. One milliliter of mother liquid was injected subcutaneously into the experimental group, and 1 mL of normal saline was injected into the control group. HE staining was performed on the skin and subcutaneous tissue at the injection site on days 10 and 30.

#### 2.2.4. CFA-Induced inflammatory pain

*The Modeling Method*: After anesthetizing, rats were placed on the operating table, and 0.1 mL of CFA solution (Sigma) was slowly injected, subcutaneously, into the left posterior plantar of the rats. The rats were then placed in separate cages.

#### 2.2.5. Measurement of mechanical pain threshold

**Figure 2.**
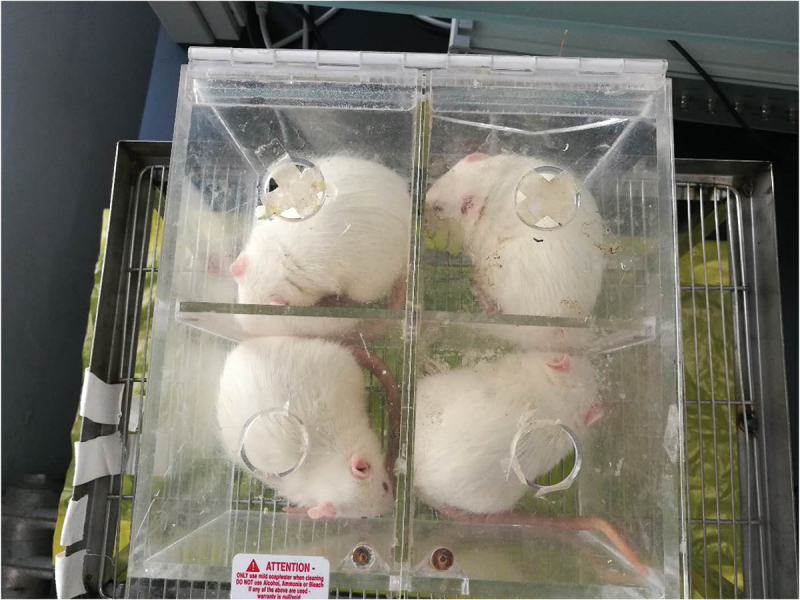
Photograph demonstrating the four grids plexiglass fixer used for the study.

*Measure Time and Environment*: Pain threshold was measured in the left posterior sole using a Von Frey filament (on days 2, 4, 6, 8, and 10 after injection). The range of time was between 15:00–17:00 everyday . The rats were put into a removable four grids plexiglass fixer (Figure 2), 30 minutes prior to the experiment, for acclimatization to the environment.

Experimental Details: 12 point, double-spaced. References are superscripted and appear after the punctuation **[**13**]**.

*Von Frey Filament Measurement Method*: The classic von Frey filaments were used to measure the mechanical pain threshold. The order of measurement was 2.0 g, 4.0 g, 6.0 g, 8.0 g, 10.0 g, 15.0 g, and 26 g. The measurement started from 2.0 g, and each type of filament was measured 5 times. The measurement site was the left posterior plantar of the rats, lasting for 5 s each time, and the interval between the two stimuli was 10s. During the procedure, it was appropriate to bend the filaments to a near rectangle. Positive results suggested an evasive response, such as foot contraction and foot licking, to the filaments. A negative result suggested that the rats did not respond to filaments. Positive results were recorded as ‘X’ and the negative results were recorded as ‘O.’

#### 2.2.6. Pain model assessment

*Local Pathology*;Two model rats were randomly selected and on the 10^th^ day after CFA injection. 0.3 cm – 0.5 cm of the skin was taken from the left posterior plantar and fixed with neutral formalin, followed by HE staining.

*Evaluation of Appearance of the Affected Feet*:Record numbers of inflammation, red and/or swollen appearance rats. For the study, rats were grouped by the envelope method, where the evaluators did not know the situation, and the same person scored the animals from beginning to end.

## 3. Statistical methods

The microsphere characterization data were analyzed using Excel 2010 software. In vitro release curves of microparticles with different feed ratios were fitted.

The biocompatibility data were analyzed using SPSS 17.0 software. For normally distributed data, multiple sets of data such as blood count, volume, and leukocyte classification were tested using one-way ANOVA and paired t-test, respectively. The chi-square test was used for comparison between counting samples in pairs and 95% confidence intervals (CIs) for pairwise comparisons were calculated with Bonferroni correction. The significance level (α) was set at 0.05.

## 4. Results and discussion

### 4.1. In vitro test

#### 4.1.1. Microsphere evaluation

To optimize their quality, microspheres with different feed ratios (1:3, 1:4, and 1:5) were synthesized. We evaluated the characteristics (shape, span, yield, encapsulation rate, drug loading, and magnetic field responsiveness) of the microspheres prepared with different feed ratios, described below.

**Figure 3.**
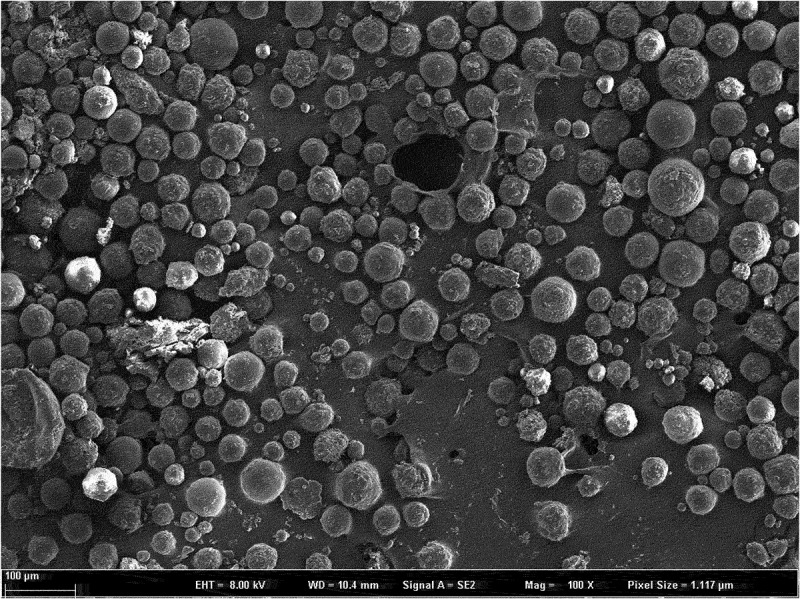
Scanning electron microscope image of microspheres prepared with a feed ratio of 1:3.

**Figure 4.**
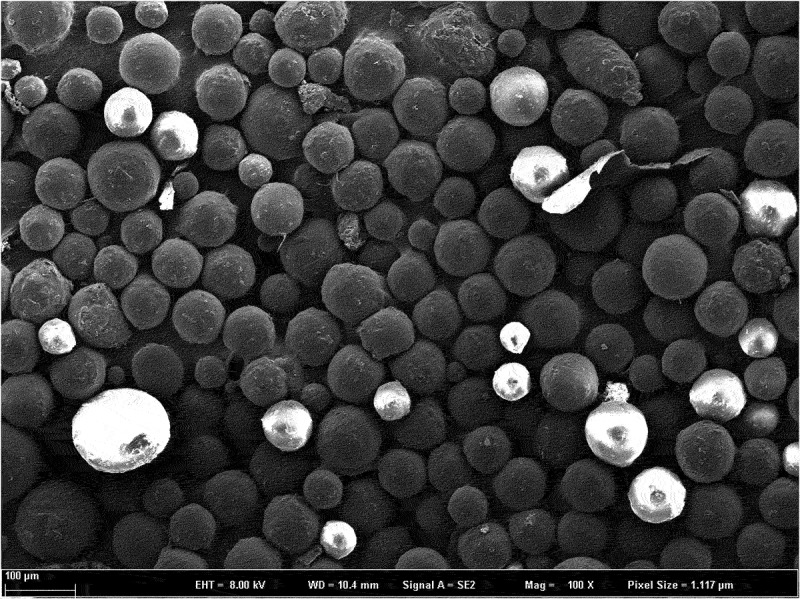
Scanning electron microscope image of microspheres prepared with a feed ratio of 1:4.

**Figure 5.**
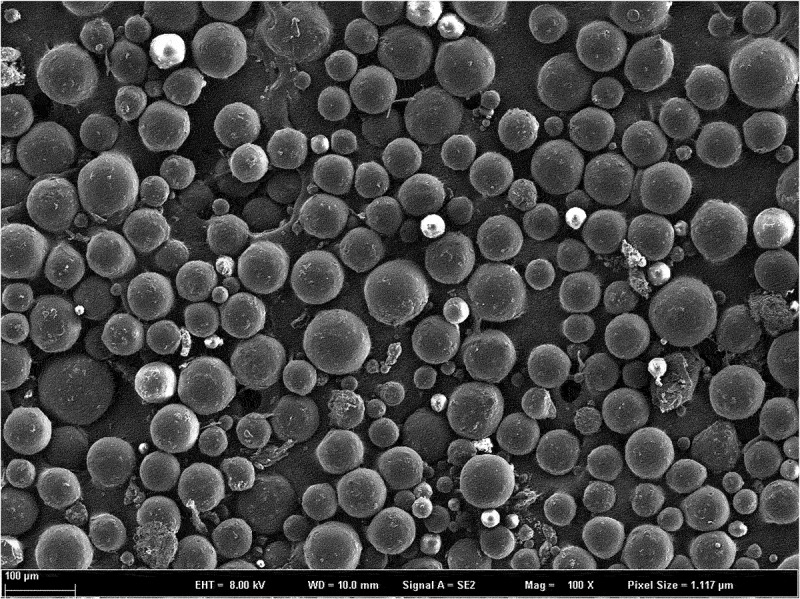
Scanning electron microscope image of microspheres, prepared with a feed ratio of 1:5.

*Characterization*: The dexamethasone-PLGA magnetic microspheres synthesized in this study are black and appeared mostly spherical under a electron microscope microscope (40 × 10) (Figure 3–5). The average diameter (span, S1) of the microspheres with feed ratios of 1:3, 1:4, and 1:5 were (27.47 ± 8.78) µm, (29.66 ± 10.02) µm, and (30.14 ± 11.2). µm, respectively. The morphology of the different microspheres, as observed under an scanning electron microscope is shown in Figures 3–5. The yield (S2) for the microspheres with feed ratios of 1:3, 1:4, and 1:5 was 87.02 ± 2.25%, 87.25 ± 3.14%, and 84.00 ± 5.53%, respectively. The encapsulation rate (S3) was 76.44 ± 3.11%, 78.89 ± 2.42%, and 71.33 ± 5.60%, respectively. The drug loading percentage (S4) was 33.09 ± 1.89%, 30.33 ± 2.01%, and 24.29 ± 1.78%, respectively. The sum values (S) for the different microspheres were 168.55 ± 3.62, 167.47 ± 4.15, and 149.48 ± 4.21, respectively. Compared with the other two groups, there was a statistically significant difference in the S value of the microspheres developed with a feed ratio of 1:5 (see Table 1 for details). This suggested that the quality of microspheres synthesized with a feed ratio of 1:3 and 1:4 is better than that of the microspheres developed with a feed ratio of 1:5. There was no significant difference in the quality of microspheres synthesized with feed ratios of 1:3 and 1:4.

As the microspheres need to be administered by a syringe, their diameter is important, and should be less than 125 µm [24]. Irrespective of the batches of microspheres used in this study, the diameters of the microspheres was found to be approximately 30 µm, significantly less than 125 µm. All batches of microspheres were able to pass smoothly through the standard 5# (0.5 mm in diameter) needle with a capacity more than 2.5 mL.

Dexamethasone is insoluble in water; therefore, the solvent evaporation method (S/O/W) was followed for microsphere synthesis. In this experiment, S (solid phase) refers to dexamethasone powder, O (oil phase) refers to dichloromethane, and W (water phase) refers to PVA. In this process, the diameter and appearance of microspheres are mainly affected by four factors: first, the diameter of the microsphere is related to the rotation speed during emulsification and volatilization. In the initial volatilization experiments, the rotational speed was 1000 r/ min, resulting in microspheres with diameters ranging from 100–150 µm, with some larger than 200 µm. Subsequent experiments performed at 1500 r /min and 1800 r /min resulted in microspheres with diameters gradually decreasing to 30 µm, which were used in this study. In addition, Zhang et. al. [8] proposed a rotational speed >10,000 r/ min for obtaining smaller diameters of microspheres. Second, the diameter and appearance of microspheres are related to the mass concentration of PLGA in the oil phase. It can affect not only the stability of the initial emulsion but also the homogeneity of the microspheres. If it is too low (1:30–1:60), the initial emulsion will be unstable, and the microspheres will have an irregular shape and uneven size. In contrast, if too high (1:10–1:15), it reduces the microsphere size but may result in irregular PLGA floc and clumpy products. Only when the PLGA mass concentration was moderate (1:20), uniform and smooth spherical microspheres could be obtained, and their diameters ranged between those synthesized with high and low PLGA mass concentrations. Third, the mass fraction of water-phase PVA also affects the diameter and appearance of the microspheres. The viscosity of the PVA solution has a certain effect on the coalescence between the initial emulsion droplets. When it is low (0.1–0.5%), the viscosity is too small to maintain the stability of the initial emulsion, and the diameter of the microspheres produced is large and non-uniform. Conversely, a high viscosity (4%) causes the fluidity of the initial emulsion to reduce significantly. Only when the PVA mass fraction was moderate (1%–2%), the diameter of the microsphere was reasonable and more uniform, meeting the experimental requirements. Fourth, the volume ratio between the oil phase and internal water phase also affects the diameter and shape of the microspheres. When the oil to water phase ratio is too low (1:1–1:2), the microspheres are not spherical, and the shell of the microspheres is loose and prone to disintegration. However, when the ratio was too high (6:1), the surface tension of the initial emulsion decreased and larger microspheres were easily formed. Only when the ratio is moderate (2:1–4:1) can the microspheres with spherical appearance and uniform size be formed. Although microspheres synthesized with a ratio of 4:1 had smaller diameter, the ones synthesized with a ratio of 2:1 were more uniform. Since the S/O/W emulsified solvent volatilization method was used in this study to prepare the microspheres, these factors have been optimized in many experiments. Finally, the parameters used in this experiment were; a volatilization revolution of 1800 r min^−1^, PLGA mass concentration of 1:20, and PVA mass fraction of 2%. The microspheres prepared under the above-mentioned conditions were found to be smooth and spherical with uniform size, good dispersivity, and a high penetration rate.

The quality evaluation of microspheres, as determined by the sum value (S), is important and related to span (S1), yield (S2), encapsulation rate (S3), and drug loading (S4). It is inversely proportional to S1 and directly proportional to S2, S3, and S4. The sum value (S) for the microspheres synthesized with the feed ratios of 1:3, 1:4 and 1:5 was found to be (168.55 ± 3.62), (167.47 ± 4.15) and (149.48 ± 4.21), respectively (Table 4). The sum value (S) for the microspheres synthesized with a feed ratio of 1:5 was significantly lower from the other two groups. There was no statistical difference between the sum value S for the microspheres synthesized with feed ratios of 1:3 and 1:4.

**Table 4.** Characteristics of dexamethasone-PLGA magnetic microspheres (*x ± s, n = 500).*

Feed ratio	Shape	Span (µm)	Yield (%)	Encapsulation rate (%)	Drug loading (%)	Sum (S)
1:3	round	27.47 ± 8.78	87.02 ± 2.25	76.44 ± 3.11	33.09 ± 1.89	168.55 ± 3.62
1:4	round	29.66 ± 10.02	87.25 ± 3.14	78.89 ± 2.42	30.33 ± 2.01	167.47 ± 4.15
1:5	round	30.14 ± 11.20	84.00 ± 5.53	71.33 ± 5.60	24.29 ± 1.78	149.48 ± 4.21*

Ps: *S value for the microspheres synthesized with a feed ratio of 1:5 was significantly different from that of other groups.

#### 4.1.2. Magnetic field responsiveness

Owing to the targeting ability of magnetic microspheres, they have been widely used in the treatment of malignant tumors, especially malignant liver tumors. For some patients with early malignant liver tumors, the treatment regimen in combination with radiotherapy shows a target-specific effect [8,25]. Guided by the magnetic field, drug-loaded microspheres can swim towards the lesion where they are gradually degraded, thereby releasing the encapsulated drugs at the lesion site. This results in maintaining a stable therapeutic concentration at the lesion site for prolonged periods of time, leading to a gradual shrinking of the immature tumors and leaving no opportunity for growth and recovery. It can also be applied to the treatment of local inflammation, as reported by Butoescu et al. [10,16,25] for the treatment of arthritis. Under the guidance of a magnetic field, the number of microspheres entering the lesion increased by 23% within 48 h compared with non-magnetic microspheres. This was then found to increase to 60% with time. Therefore, with the progress of microsphere manufacturing technology, the number and speed of magnetic microspheres that can be accumulated in lesions has been shown to increase, more convincingly in latter cases, under the guidance of a magnetic field, resulting in an enhanced therapeutic effect.

In this study, the magnetic field responsiveness of the microspheres was studied by observing the microspheres under an optical microscope, with an externally applied magnetic field strength of 4000 GS. The dexamethasone-PLGA magnetic microspheres were observed to move irregularly with their direction towards the magnet, and the motion distance was found to be approximately 80.0 ± 5.0 mm towards the bottle wall.

#### 4.1.3. Cumulative release rates

The in vitro cumulative release rates of dexamethasone-PLGA magnetic microspheres synthesized with feed ratios of 1:3, 1:4 and 1:5 were 79.12%, 32.91%, and 40.33% on the 2nd day, 82.6%, 62.74%, and 76.04% on the 7th day, and 86.34%, 66.99%, and 97.85% on the 14th day, respectively. Compared with other groups, the in vitro burst release from the microspheres synthesized with a feed ratio of 1:3 was significantly high (Table 5).

**Table 5.** In vitro cumulative release rates of microspheres synthesized with different feed ratios

Time point (d)	Feed ratios	Cumulative release rate (%)
2^nd^	1:3	79.12*
	1:4	32.91
	1:5	40.33
7^th^	1:3	82.61*
	1:4	62.74
	1.5	76.04
14^th^	1:3	86.34
	1:4	66.99
	1:5	97.85

Ps: *Compared with other groups, the in vitro release of the microspheres synthesized with a feed ratio of 1:3 was significantly high, *P < 0.01.*

### 4.2. Drug release model and linear trend in vitro

The in vitro drug release models for microspheres synthesized with different feed ratios were different. According to their release characteristics, the fitted model and linear regression were as follows.

**Figure 6.**
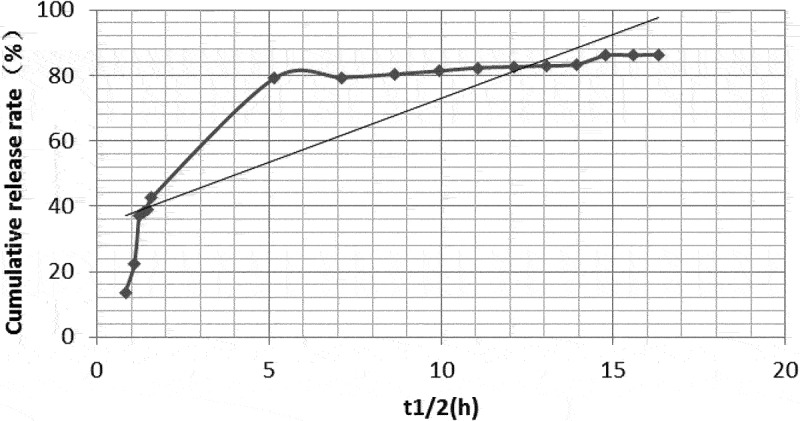
In vitro drug release linear profile of microspheres (feed ratio of 1:3).

The model equation for analyzing release profile from microspheres synthesized with a feed ratio of 1:3 was Q = 22.313In(t_1/2_) + 28.651 (R^2^ = 0.949). The linear trend of drug release was best fit into a logarithmic equation. As shown in Tables 5 and 6 and Figure 6, the drug burst release rate was 79.12% on days 1 and 2, 82.6% on day 7, and 86.34% on day 14. This indicates that the drug release profile immediately attained a stable sustained-release phase and the drug concentration reached a steady state after the burst release of the drug on the 2^nd^ day.

**Table 6.** In vitro release models and R^2^ values of microspheres synthesized with different feed ratios

Feed ratio	In vitro release models	R^2^
1:3	Q = 22.313In(t_1/2_) + 28.651	0.949
1:4	Q = 4.0844(t_1/2_) + 6.6602	0.977
1:5	Q = 5.212(t_1/2_) + 12.481	0.975

**Figure 7.**
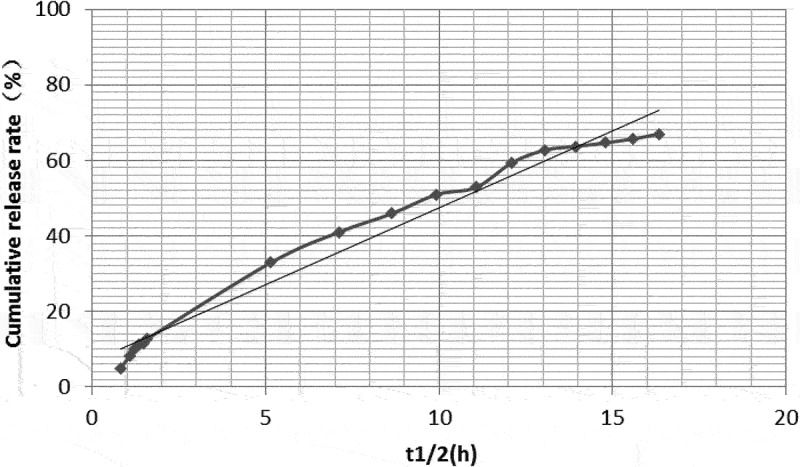
In vitro drug release linear profile of microspheres (feed ratio of 1:4).

The model equation for analyzing release profile from microspheres synthesized with a feed ratio of 1:4 was Q = 4.0844(t_1/2_) + 6.6602, (R^2^ = 0.977). Its linear trend was a best fit to the zero-order kinetic model. As shown in Tables 5 and 6 and Figure 7, the drug burst release rate was 32.91% on days 1 and 2, 62.74% on day 7, and 66.99% on day 14.

**Figure 8.**
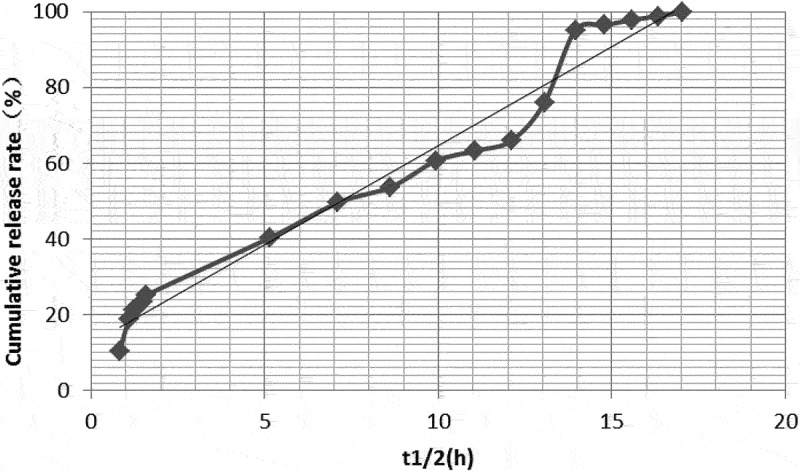
In vitro drug release linear profile of microspheres (feed ratio of 1:5).

The model for analyzing release profile from microspheres synthesized with a feed ratio of 1:5 was Q = 5.212(t_1/2_) + 12.481 (R^2^ = 0.975). Its linear model is more consistent with first-order kinetics. As shown in Tables 5 and 6 and Figure 8, the burst release rate of the drug was 40.33% on days 1 and 2, 76.04% on day 7, and 97.85% on day 14.

In vitro degradation of PLGA microspheres and subsequent drug release is mainly divided into two phases; burst- and sustained-release phases. An ideal microsphere is characterized by a long sustained-release time, small burst release amount, and high drug loading [16,26]. According to the drug loading analysis in the previous part of this study, the drug loadings of microspheres synthesized with a feed ratios of 1:3, 1:4, and 1:5 were 33.09 ± 1.89%, 30.33 ± 2.01%, and 24.29 ± 1.78%, respectively. In terms of drug loading, the microspheres, synthesize with a feed ratio of 1:5, showed a significantly lower drug loading capacity as compared with the other two groups. There was no significant difference between the drug loading of microspheres synthesized with feed ratios of 1:3 and 1:4. In terms of the burst release phase, the cumulative release rate, in vitro, on the 2^nd^ day was 79.12%, 32.91%, and 40.33%, respectively, for the microspheres synthesized with a feed ratio of 1:3, 1:4 and 1:5. The above data suggested that the drug release profile of microspheres, synthesized with a feed ratio of 1:3 was significantly higher as compared with the other two groups. The reason for the sudden phase release may be that during the wrapping material selection and production process of the microsphere, some drug molecules were not completely wrapped in the microsphere and were adsorbed on the surface, near the surface, or around the internal cavity of the microsphere. Therefore, in the early stages, a sudden release of the drug was observed. In addition, the quality and purity of PLGA, the preparation method of the microsphere, instrument, and synthesis parameters may also have an effect [16]. Zolnik et al. [27–30] suggested that differences in in vitro cumulative release rates could be caused by different rates and degrees of degradation of different PLGA types. Different PLGA/PLA ratios (e.g., 85/15, 75/25, 50/50, etc.) have been reported to degrade at different rates, as shown by Dobrovolskaia et al. [31], who reported that when the PLGA content was high, the degradation rate of microspheres was higher than that synthesized with a higher PLA content. However, as PLA has poor hydrophilicity, combination of the two could provide a polymer with optimum degradation rates and hydrophobicity, and PLGA/PLA ratios of 75/25 and 50/50 have been shown to be better for microsphere synthesis and drug loading. In the preliminary studies, it was found that the diameter of microspheres synthesized with 75/25 PLGA/PLA was approximately 100 µm, whereas the diameter of the microspheres synthesized with 50/50 PLGA/PLA was approximately 30 µm. However, this does not consider the production process, and the quality of microspheres gradually improved.

As stated earlier, the degradation patterns were found to be different for microspheres synthesized with different feed ratios. Although the microspheres synthesized with a feed ratio of 1:3 showed some sustained release and high drug loading, the sudden burst release phase was significantly too high. The cumulative release for this group of microspheres reached 79.12% of the drug loading on day 2. The release of drugs in the sudden release phase was mainly due to the dissolution mechanism and direct diffusion. This approach is commonly seen in non-sustained release of drugs [32]. However, most of microspheres with obvious sudden exploding have poor sustained-release properties; therefore, they are not ideal microspheres.

The in vitro drug release model of the microspheres synthesized with a feed ratio of 1:5 was close to a zero-order release curve. On day 2, 40.33% of the drug was released in the burst release phase, and 76.04% and 97.85% were released cumulatively on days 7 and 14, respectively. In other words, 35.71% and 21.81% of the drug was released during days 2–7 and 7–14, respectively. In conclusion, the drug burst release in this group was not significantly high and the sustained release was good. However, compared with the other groups, the drug loading of the microspheres in this group was only 24.29 ± 1.78%, which was the lowest of the three groups. Therefore, it was not considered an ideal microsphere formulation.

The in vitro drug release equation of microspheres synthesized with a feed ratio of 1:4 corresponds to a zero-order kinetic model, suggesting that a drug was released at a constant rate over time. On day 2, the burst release phase for this group showed a release of 32.91% of the drug, corresponding to nearly 1/3. This was followed by a gradual slowdown in the drug release. On day 14, 66.99% was released, suggesting a release of 1/3 from days 2 to 14. The drug loading of this group was 30.33 ± 2.01%, and the sum value (S) of the microsphere quality score (167.47 ± 4.15) was significantly better than that of the microspheres synthesized with a feed ratio of 1:5 (149.48 ± 4.21). Therefore, the dexamethasone-PLGA magnetic microspheres synthesized with a feed ratio of 1:4 were considered to be superior to other microspheres in terms of its burst release, sustained release, drug loading, yield, encapsulation rate, span, and other aspects.

#### 4.1.4. Microsphere stability

Microsphere stability was studied by storing the microspheres in a refrigerator at 4–8 °C, for 3 months. The drug loading, particle size, and other physical properties did not change significantly in 3 months, suggesting good stability (Table 7).

**Table 7.** Stability tests of microspheres with different feed ratios (N ≥ 500)

Feed ratio	Time points (month)	Drug loading (%)	Span (µm)
1:3	0	33.09 ± 1.89	27.47 ± 8.78
	1	32.26 ± 2.01	28.01 ± 8.92
	2	33.28 ± 1.23	26.32 ± 7.33
	3	32.67 ± 1.66	29.11 ± 9.15
1:4	0	30.33 ± 2.01	29.66 ± 10.02
	1	31.26 ± 2.45	31.24 ± 10.87
	2	30.78 ± 1.96	28.99 ± 9.86
	3	31.71 ± 2.34	30.58 ± 10.29
1:5	0	24.29 ± 1.78	30.14 ± 11.20
	1	25.31 ± 2.04	30.55 ± 10.26
	2	26.77 ± 3.20	31.29 ± 11.83
	3	25.36 ± 2.11	31.54 ± 11.90

#### 4.1.5. Biocompatibility

As previously described, blank microspheres were synthesized using the same process as that of dexamethasone-PLGA magnetic microspheres with a feed ratio of 1:4 to evaluate the biocompatibility. The blood compatibility and histocompatibility are discussed below.

#### 4.1.6. Hemolysis test

For the in vitro hemolysis test, samples with concentrations of 1 mg/ mL, 2 mg/ mL, and 4 mg/ mL were tested and the absorbance was found to be 0.006, 0.011 and 0.032. The hemolysis rate was calculated to be 0.5%, 1.2%, and 3.3% for the three concentrations tested. The absorbance of negative control and positive control was 0.003 and 0.802 corresponding to a hemolysis rate of 0% and 99.9%, respectively). A hemolysis rate of less than 5% is considered standard for a sample with no hemolysis effect. No hemolysis effect was observed for the different concentrations studied. (Table 8)

**Table 8.** Absorbance and hemolysis rate of samples (%, n = 3)

Samples	Hemolysis rate (%)	Absorbance
1 mg/ mL	0.5	0.006
2 mg/ mL	1.2	0.011
4 mg/ mL	3.3	0.032
negative control	0	0.003
positive control	99.9	0.802

#### 4.1.7. Routine Blood Examination

The effects of different doses of microspheres on routine blood count in rats were as follows: *WBC, RBC, and PLA* counts: The changes in WBC counts, RBCs, and platelet counts of rats in the high, medium, and low dose groups at 0.5 h, 6 h, 12 h, and 24 h after injection were all within the normal range, [33]and the changes were not significantly different to the values obtained prior to the treatment, as detailed in Table 9.

**Table 9.** Effects of different doses of microspheres on blood cell count

Indicators	Doses (mL /100 mg)	Time points				
		Before dosing	0.5 h	6 h	12 h	24 h
RBC counts	0.2	6.50	6.26	5.54	6.19	6.88
(×10^12^ L^−1^)	0.5	7.33	7.46	7.86	8.21	8.60
	0.8	5.96	6.16	6.83	5.78	6.32
WBC counts	0.2	6.11	5.05	6.35	7.17	7.48
(×10^9^ L^−1^)	0.5	8.29	9.02	8.88	9.12	9.34
	0.8	9.66	7.90	10.00	8.92	9.74
Platelet counts	0.2	717.8	754.2	798.8	813.0	809.3
(×10^9^ L^−1^)	0.5	851.3	899.0	936.5	797.5	864.4
	0.8	665.6	716.2	724.3	687.1	703.9

*Volume of RBC and PLA*: The changes in RBCs and platelets at 0.5 h, 6 h, 12 h, and 24 h after injection were all within the normal range [34,35], and the changes were not significantly different as compared with those obtained prior to the treatment, as detailed in Table 10.

**Table 10.** Effect of different doses of microspheres on blood cell volume

Indicators	Doses (mL /100 mg)	Time points				
		Before dosing	0.5 h	6 h	12 h	24 h
Mean erythrocyte volume	0.2	67.8	63.5	60.6	59.8	63.9
(fl)	0.5	55.3	60.2	58.1	63.2	66.3
	0.8	52.6	52.0	55.5	60.2	58.6
Mean platelet volume	0.2	7.1	6.5	7.4	8.1	7.0
(fl)	0.5	7.7	7.5	7.8	8.3	8.1
	0.8	662.5	716.2	701.0	695.6	688.3

*Leukocyte Classification (%)*: The changes in neutrophils (%) were not significantly different 0.5 h after injection, but were found to be significantly decreased 6 h, 12 h, and 24 h after injection. However, the change in lymphocytes (%) was found to be reversed. The change in lymphocyte percentage was not significantly different 0.5 h after injection, but it was significantly increased at 6 h, 12 h, and 24 h post-treatment. Monocytes (%) showed statistically significant changes at 12 h and 24 h post-treatment. Eosinophils (%) were significantly reduced at 0.5 h, 6 h, 12 h, and 24 h after injection. Additionally, the changes in basophils were not statistically significant, as detailed in Table 11.

**Table 11.** Effect of different doses of microspheres on leukocyte classification

Indicators	Doses (mL /100 mg)	Time points				
		Before dosing	0.5 h	6 h	12 h	24 h
Neutrophils (%)	0.2	32.3	33.6	26.1**	15.9**	11.0**
	0.5	39.5	40.3	22.2**	10.1**	9.7**
	0.8	26.3	30.3	14.0**	8.3**	6.3**
Lymphocytes (%)	0.2	58.4	59.9	63.4*	72.7**	77.4**
	0.5	49.3	51.2	68.6**	68.6**	75.4**
	0.8	64.8	63.4	81.0**	81.2**	82.2**
Monocytes (%)	0.2	6.0	5.1	7.2	8.6*	9.7*
	0.5	7.7	6.5	8.4	9.9*	11.2*
	0.8	6.3	5.2	4.1	8.2*	9.1*
Eosinophils	0.2	3.3	1.4**	1.0**	0.9**	1.5**
(%)	0.5	3.5	2.0**	0.8**	1.5**	1.9**
	0.8	2.6	1.1**	0.9**	1.8**	2.0**
Basophils	0.2	0	0	0	0.2	0.4
(%)	0.5	0	0	0	0.3	0.5
	0.8	0	0	0	0.5	0.4

Ps: * P < 0.05, ** P < *0.01*

### 4.2. In vivo tests

#### 4.2.1. Histocompatibility

**Figure 9.**
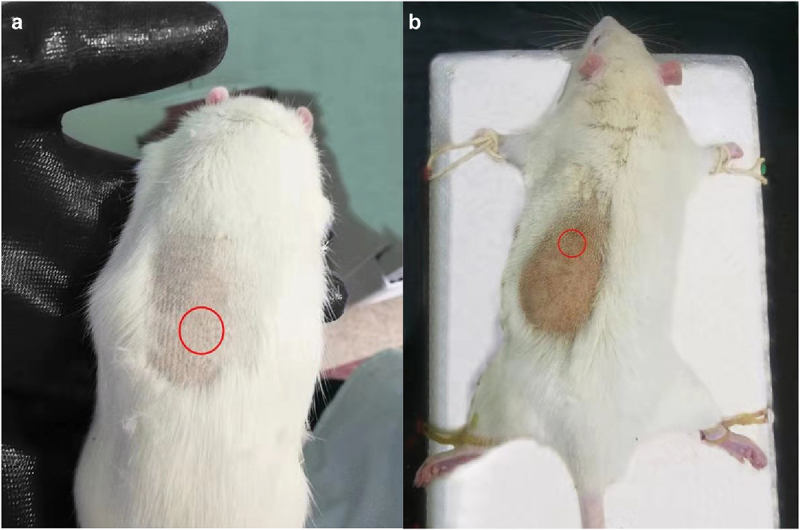
Appearance of skin on the site where microspheres were subcutaneously injected. The skin of rats showed no signs of redness, swelling, ulceration, and infection on the 10th (a) and 30th (b) days, post-injection.

*Skin Appearance*: After subcutaneous injection of microspheres, the skin of rats showed no signs of redness, swelling, ulceration, or infection on days 10 and 30, post-injection (Figure 9).

**Figure 10.**
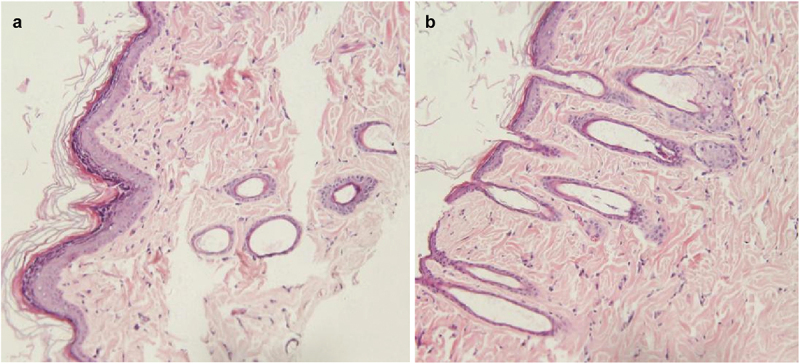
HE staining of the skin at injection sites. The HE staining of skin sections obtained from the injection site showed no difference on days 10 (a) and 30 (b) after subcutaneous injection compared to the normal skin.

*Pathological sections of the injection site of rats (HE staining*);The HE staining of the skin sections obtained from the injection site showed no difference on days 10 and 30 after subcutaneous injection compared to the normal skin. (Figure 10).

The results of biocompatibility experiments are a key indicator of whether microspheres can be successfully applied in clinical treatment. The blood compatibility test and histocompatibility test are more intuitive and maneuverable than the observations made at the cellular level. Factors that can influence biocompatibility and histocompatibility are particle size, shape, and concentration of microspheres.

Kotla et al. [36,37] showed that the hemolysis rate of microspheres at the same concentration was inversely proportional to the particle size. In particular, microspheres with diameters less than 10 µm are more likely to be engulfed by macrophages and peripheral giant cells, promoting their accelerated degradation [34]. The diameter of the microspheres synthesized in this study was approximately 25–30 µm, which cannot be easily taken up and has certain slow-release performance. However, the penetration rate of microspheres with large particle size was low and their clinical use has been limited. Hence, the size of the microspheres should not be too large or too small. In addition, the shape (roundness) of the microspheres is also highly correlated with the hemolysis rate [38]. Angular materials are more likely to scratch red blood cells, causing them to rupture leading to hemolysis, whereas smooth, rounded microspheres are more compatible.

The concentration of microspheres affects the hemolysis rate. Previous studies [34] showed that the hemolysis rate of microspheres with a concentration of 1.5 mg/mL was lower than that of other groups. This effect may be due to tension or osmotic pressure. In this study, none of the microspheres tested resulted in a hemolysis rate of more than 5%, regardless of its concentration, suggesting that the above concentrations of microspheres are in line with national standards. This may be due to the biocompatibility of PLGA, which can be slowly degraded in vivo, and the degradation products have no adverse effects on rats. In addition, the high stability of PLGA also prevents cell damage caused by sudden drug release [7,8].

As shown in the above experimental data, the microspheres produced in our experiment had no significant effect on the blood count and volume of rats in the blood compatibility experiments. However, it had an influence on the percentage of white blood cell subtypes. As shown in Table 12, lymphocytes were elevated approximately 6 h after microsphere injection, whereas neutrophil count was decreased. Monocytes (%) increased significantly approximately 12 h after injection, and eosinophils (%) decreased gradually. All the above phenomena are precisely related to the invasion of microspheres into the body as foreign bodies, and leukocyte mobilization and phagocytosis activities are closely related. Galluzzi L [39]and Martinod K [40] also mentioned this phenomenon, but it could not be used to explain its poor biocompatibility. Instead, it was the normal reaction of the body to foreign bodies. In this experiment, the skin appearance and HE staining analysis of rats on days 10 and 30 post-injection were similar to that observed for normal rat tissues.

**Table 12.** The number of inflammation spots in the CFA-induced chronic inflammatory pain model rats after treatment (n = 6)

	PC	OMT	MD	MD + MT	PD	PD + MT
2nd day	6	6	6	6	4	4
4th day	6	6	5	4	4	4
6th day	6	6	2	1	3	2
8th day	5	5	1	0	1	1
10th day	5	5	0	0	0	1

#### 4.2.2. Chronic plantar inflammatory model

The pain threshold before CFA injection was 22.2 ± 4.3, whereas the pain threshold on days 2, 4, 6, 8, and 10 after CFA injection was 7.9 ± 1.2, 6.0 ± 0.8, 3.3 ± 0.5, 4.1 ± 1.6, and 3.1 ± 0.6, respectively. Compared with the mechanical pain threshold of rats before CFA administration, the pain threshold of rats 2–10 days after administration was significantly lower (Figure 12).

**Figure 11.**
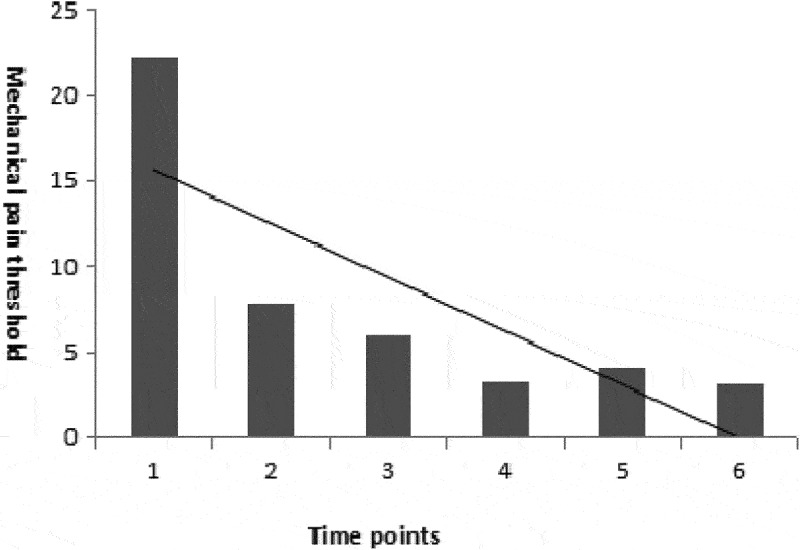
CFA mechanical pain threshold in rats (n = 40).

**Figure 12.**
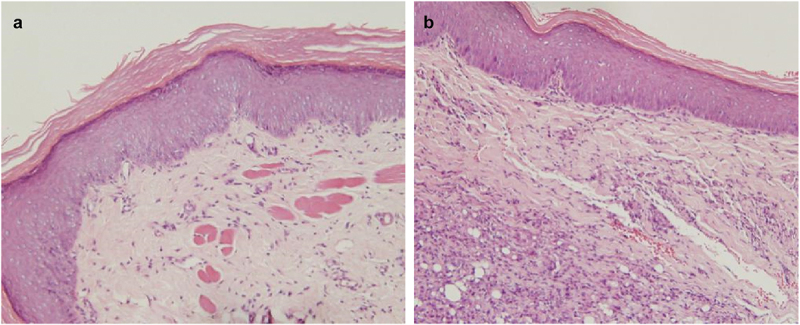
HE staining of the skin at injection sites. (a) normal plantar tissue; (b) model mouse plantar tissue, showing an inflammatory response.

Time point 1: before administration; Time point 2: 2 days after administration; Time point 3: 4 days after administration; Time point 4: 6 days after administration; Time point 5: 8 days after administration; and Time point 6: 10 days after administration.

Ps: Compared with pre-administration, the threshold of mechanical pain was significantly reduced after administration (P < 0.01).

Pathological section (HE staining): On the 10th day post CFA injection into the bottom of the left hind limb of rats, a large number of inflammatory cells were observed in the HE stained sections, also characterized by a disordered structure (Figure 12).

CFA can induce local acute and chronic inflammation, and the inflammatory model is characterized by elevated T cells and CII antibodies, suggesting that both cellular and humoral immunity are involved [41]. However, to establish that the CFA-induced pain model meets the needs of chronic inflammation modeling required in this study, we injected 0.1 mL of CFA in the rat plantar. On the 2nd day after injection, the skin temperature of the infected rats increased significantly. The swelling of the affected foot was significantly higher than that of the healthy side. The activity of the rats was significantly reduced. In some rats, foot licking activity was also increased.

Compared with the mechanical pain threshold before CFA injection, the mechanical pain threshold between days 2 and 10 after CFA injection was significantly lower than that before CFA injection, and was found to be the lowest on the 6th day (Figure 11). A large number of inflammatory cells infiltrated into the skin and soft tissue of the lateral foot, as observed in the HE stained section, and the tissue structure was also found to be disordered. These are typical features of chronic inflammation [29]. Therefore, it was concluded that the CFA rat plantar injection model meets the requirements of this experiment.

#### 4.2.3 Post-treatment manifestations

*Appearance*: Before treatment, there was no significant difference in the appearance of inflammation in each group. On 2nd day, the number of red swelling in phosphate dexamethasone (PD with/without MT) group was less than that in the microsphere group (MD with/without MT), On the 6th day, the number of red swelling in the microsphere dexamethasone + magnetic therapy group (MD + MT) was less than that of other groups (Table 12), details of experimental group were showed in Table 2.

**Figure 13.**
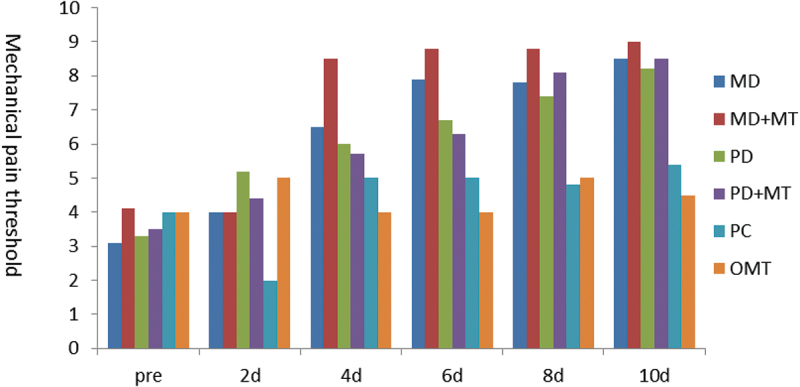
Mechanical pain threshold before and after treatment.

*Mechanical pain threshold*: Compared with before treatment, except for the positive control group (PC) and magnetic therapy group (MT), the other groups showed significant improvement on days 4 and 6. The pain threshold of the microsphere + magnetic therapy group (MD + MT) was significantly higher than that of the other groups, as studied on days 4 and 6. The pain threshold of the microsphere group (MD) and the microsphere + magnetic therapy group (MD + MT) on day 8 was significantly higher than that of the other groups (Figure 13).

The above results suggested that the therapeutic effect of the MD group was similar to that of the PD group, but the effect of microspheres was enhanced when combined with magnetic therapy.

**Figure 14.**
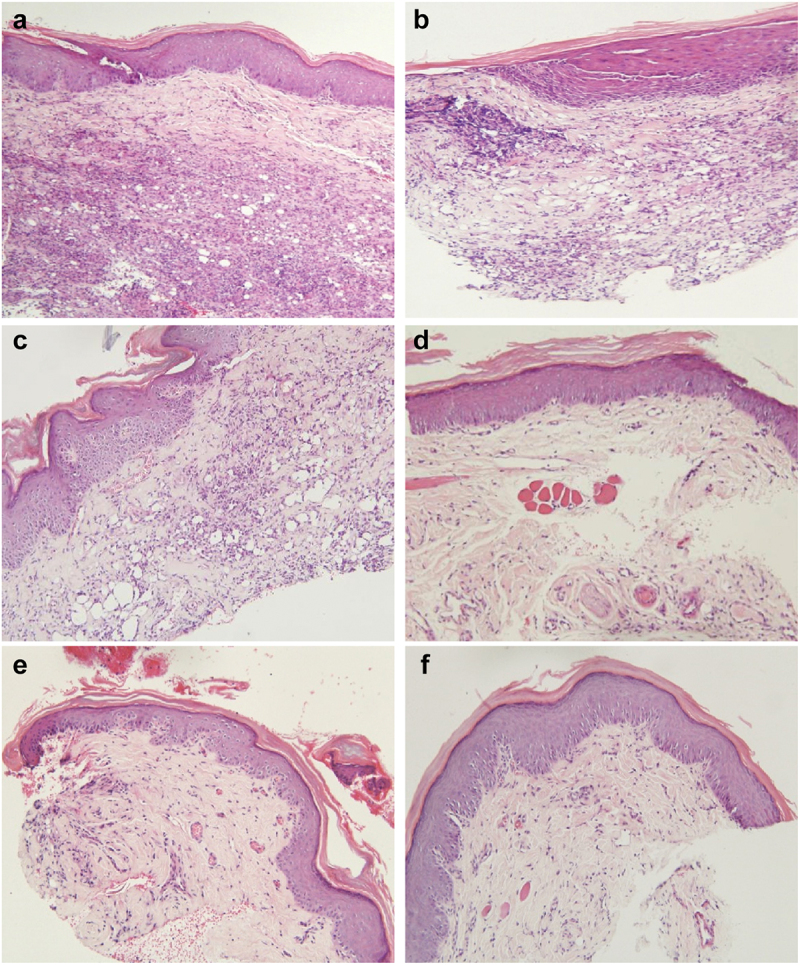
HE staining of plantar 10 days after therapy: A: positive contrast group (PC), B: only magnetic treatment(OMT), C: dexamethasone microspheres therapy (MD), D: dexamethasone microspheres + magnetic therapy(MD+MT). E: dexamethasone phosphate therapy (PD), F:dexamethasone phosphate + magnetic therapy(PD+MT)

*Pathological section (HE staining*): Compared with the control group (PC and OMT; Figure 14a,b and Figure 15a,b), dexamethasone-treated groups (MD and PD;) and combination magnetic therapy groups (MD + MT, PD + MT;) showed a reduction in inflammatory cells in the pathological sections (HE 200×) of the affected feet 10 days after treatment.

According to the appearance, mechanical pain threshold, and histological analysis, the effect on rats in the MD + MT group was better than PD groups after the 4th day, PD groups worked better in the first 3 days.

Because we designed the microspheres to test the effects of different dosing methods, the microspheres are packed with the same dose of dexamethasone as a single dosing. In the single-dose administration group, the initial local injection dose was larger and the drug concentration was higher, so the initial effect was better. In the late stage, local drug concentration attenuated quickly and the effect was not good. It is well known that high burst release batches should be excluded when selecting excellent microspheres. Therefore, some batches of dexamethasone microspheres were not selected for its high burst release. It was normal that the local drug concentration in the microsphere group was lower than that in the single dose group. However, due to its slow release, it can keep the local drug concentration stable all the time, and its early effect is slightly worse than that of the single dose administration group. The aftereffects are better (7–10 days). In addition, under the intervention of magnetic field, the magnetic particles in the microsphere vibrate at the affected area to produce micro current and heat energy, which speeds up local circulation and assists better absorption of drugs. But when used alone, its therapeutic effect is limited In conclusion, the above treatment methods have advantages and disadvantages, but if the treatment can be integrated with the above treatment methods can maximize the advantages and avoid the disadvantages. The treatment regimen consists of a single dose combined with magnetic microspheres and assisted magnetic field therapy. As a result, multimodal treatment is the best choice for reducing drug use and amplifying efficacy.

## References

[1] Borisovskaya A, Chmelik E, Karnik A. Exercise and chronic pain. Adv Exp Med Biol. 2020;1228:233–253. 32342462 10.1007/978-981-15-1792-1_16

[2] Foley HE, Knight JC, Ploughman M, et al. Association of chronic pain with comorbidities and health care utilization: a retrospective cohort study using health administrative data. PAIN. 2021;162(11):2737–2749. articles in press. 33902092 10.1097/j.pain.0000000000002264

[3] Valencia Moya A, Navarro Suay R, Fernández González JA, et al. Selective local anesthesia versus corticosteroid infiltration on low back pain: a randomized clinical trial[J]. Revista Española de Anestesiología y Reanimación (English Edition). 2020;67(1):1–7. 10.1016/j.redar.2019.08.005 31776012

[4] Shanthanna H, Busse J, Wang L, et al. Addition of corticosteroids to local anaesthetics for chronic non-cancer pain injections: a systematic review and meta-analysis of randomised controlled trials. Br J Anaesth. 2020;125(5):779–801. 32798067 10.1016/j.bja.2020.06.062

[5] Tryfonidou MA, de Vries G, Hennink WE, et al. “Old Drugs, New Tricks” - local controlled drug release systems for treatment of degenerative joint disease. Adv Drug Deliv Rev. 2020;160:170–185. 33122086 10.1016/j.addr.2020.10.012

[6] Bhatia A, Bril V, Brull RT, et al. Study protocol for a pilot, randomised, double-blinded, placebo controlled trial of perineural local anaesthetics and steroids for chronic post-traumatic neuropathic pain in the ankle and foot: the PREPLANS study. BMJ Open. 2016;6(6):e012293. 10.1136/bmjopen-2016-012293 PMC4932328 27334885

[7] Zhang W, Xu W, Ning C, et al. Long-acting hydrogel/microsphere composite sequentially releases dexmedetomidine and bupivacaine for prolonged synergistic analgesia. Biomaterials. 2018;S0142961218305362. doi:10.1016/j.biomaterials. 30099261

[8] Zhang YJ, Yin QQ, Gong DY, et al. The preclinical pharmacological study of a novel long-acting local anesthetic, a fixed-dose combination of QX-OH/Levobupivacaine, in rats. Front Pharmacol. 2019;10:10. 31474859 10.3389/fphar.2019.00895 PMC6704344

[9] Nienaber CA, Ratib O, Gambhir SS, et al. A quantitative index of regional blood flow in canine myocardium derived noninvasively with N-13 ammonia and dynamic positron emission tomography. J Am Coll Cardiol. 1991;17(1):260–269. 1987234 10.1016/0735-1097(91)90736-s

[10] Nicoleta B, Seemayer, C. A., Foti M, et al. Dexamethasone-containing PLGA superparamagnetic microparticles as carriers for the local treatment of arthritis. BIOMATERIALS. 2009;30(9):1772–1780. 19135244 10.1016/j.biomaterials.2008.12.017

[11] Ratzinger G, Wang X, Wirth M, et al. Targeted PLGA microparticles as a novel concept for treatment of lactose intolerance. J Control Release. 2010;147(2):187–192. 20435074 10.1016/j.jconrel.2010.04.017

[12] Galeska I, Kim TK, Patil SD, et al. Controlled release of dexamethasone from PLGA microspheres embedded within polyacid-containing PVA hydrogels. AAPS J. 2005;7(1):E231–40. 16146344 10.1208/aapsj070122 PMC2751512

[13] Gu B, Burgess DJ. Prediction of dexamethasone release from PLGA microspheres prepared with polymer blends using a design of experiment approach. Int J Pharm. 2015;495(1):393–403. 26325309 10.1016/j.ijpharm.2015.08.089 PMC4609624

[14] Villanueva JR, Bravo-Osuna I, Herrero-Vanrell R, et al. Optimising the controlled release of dexamethasone from a new generation of PLGA-based microspheres intended for intravitreal administration[J]. European journal of pharmaceutical sciences: official journal of the European Federation for Pharmaceutical Sciences. Eur J Pharm Sci. 2016;92287–297. doi:10.1016/j.ejps.2016.03.012. 26987610

[15] Zolnik BS, Burgess DJ. Evaluation of in vivo-in vitro release of dexamethasone from PLGA microspheres. J Control Release. 2008;127(2):137–145. 18282629 10.1016/j.jconrel.2008.01.004

[16] Butoescu N, Jordan O, Burdet P, et al. Dexamethasone-containing biodegradable superparamagnetic microparticles for intra-articular administration: physicochemical and magnetic properties, in vitro and in vivo drug release. Eur J Pharm Biopharm. 2009;72(3):529–538. 19303928 10.1016/j.ejpb.2009.03.003

[17] Widder K, Flouret G, Senyei A. Magnetic microspheres: synthesis of a novel parenteral drug carrier. J Pharm Sci. 2010;68(1):79–82. 10.1002/jps.2600680124 569198

[18] Gongqin Y, Guanlin Z, Fei H. Solvothermal synthesis of single-crystal magnetite hollow sub-microspheres: a novel formation mechanism and magnetic properties. Aus J Chem. 2016;69(7):798–804.

[19] Chang M, Smith S, Thorpe A, et al. Evaluation of phenoxybenzamine in the CFA model of pain following gene expression studies and connectivity mapping. Mol Pain. 2010;6(1):56. 20846436 10.1186/1744-8069-6-56 PMC2949723

[20] Peng X, Yang L, Heng Z, et al. Surface modification by microencapsule coating and grafting to prepare high hydrophilic polytetrafluoroethylene micropowder. React Funct Polym. 2021;167:105029.

[21] Xiao WA, Zhuang DA, Yz A, et al. Effects of lutein particle size in embedding emulsions on encapsulation efficiency, storage stability, and dissolution rate of microencapsules through spray drying. Lwt. 2021;146(7):111430.

[22] Samira B, Donya, Bagheri, S., Abd Hamid, S. B., et al. Progress in electrochemical synthesis of magnetic iron oxide nanoparticles. J Magn Magn Mater. 2014;368(40):207–229.

[23] Ménager C, Sandre O, Mangili J, et al. Preparation and swelling of hydrophilic magnetic microgels. POLYMER. 2004;45(8):2475–2481.

[24] Sampath SS, Garvin K, Robinson DH. Preparation and characterization of biodegradable poly(l-lactic acid) gentamicin delivery systems. Int J Pharm. 1992;78(2–3):165–174.

[25] Xie M, Gong W, Kong L, et al. Solution-processed whispering-gallery-mode microsphere lasers based on colloidal CsPbBr3 perovskite nanocrystals. NANOTECHNOLOGY. 2022 8;33(11):115204. 10.1088/1361-6528/ac4131 34879353

[26] Formica FA, Barreto G, Zenobi-Wong M. Cartilage-targeting dexamethasone prodrugs increase the efficacy of dexamethasone. J Control Release. 2018;295:118–129. 30572035 10.1016/j.jconrel.2018.12.025

[27] Zolnik BS, González-Fernández S, Ndobrovolskaia MA. Nanoparticles and the immune system. Endocrinology. 2009;151(2):458–465. 20016026 10.1210/en.2009-1082 PMC2817614

[28] Zolnik BS, Stern ST, Kaiser JM, et al. Rapid distribution of liposomal short-chain ceramide in vitro and in vivo. Drug Metab Dispos. 2008;36(8):1709–1715. 18490436 10.1124/dmd.107.019679

[29] Zolnik BS, Burgess DJ. Evaluation of in vivo-in vitro release of dexamethasone from PLGA microspheres. J Control Release. 2008;127(2):137–145. 18282629 10.1016/j.jconrel.2008.01.004

[30] Zolnik BS, Burgess DJ. Effect of acidic pH on PLGA microsphere degradation and release. J Control Release. 2007;122(3):338–344. 17644208 10.1016/j.jconrel.2007.05.034

[31] Dobrovolskaia MA, Shurin M, Shvedova AA. Current understanding of interactions between nanoparticles and the immune system . Immune Aspects of Biopharmaceuticals and Nanomedicines. 2019;299:78–89. doi:10.1016/j.taap.2015.12.022. PMC4811709 26739622

[32] Hinda Ezzaier JAM, Cyrille Claudet GH, Kuzhir Osa P. Kinetics of aggregation and magnetic separation of multicore iron oxide nanoparticles: effect of the grafted layer thickness. J Nanomater. 2018;8:8. 10.3390/nano8080623 PMC6116255 30126110

[33] Gifford SC, Strachan BC, Hui X, et al. A portable system for processing donated whole blood into high quality components without centrifugation. PLoS One. 2018;13(1):e0190827. 29346441 10.1371/journal.pone.0190827 PMC5773086

[34] Tomic I, Vidis-Millward A, Mueller-Zsigmondy M, et al. Setting accelerated dissolution test for PLGA microspheres containing peptide, investigation of critical parameters affecting drug release rate and mechanism. Int J Pharm. 2016;505(1–2):42–51. 27025293 10.1016/j.ijpharm.2016.03.048

[35] Kozlowska J, Stachowiak N, Sionkowska A. The preparation and characterization of composite materials by incorporating microspheres into a collagen/hydroxyethyl cellulose matrix. Polym Test. 2018;69:S0142941818300497.

[36] Zhang M, Hong Y, Chen W, et al. Polymers for DNA vaccine delivery. ACS Biomater Sci Eng. 2017;3(2):108–125. doi:10.1021/acsbiomaterials.6b00418. 33450790

[37] Reddy KS, Rashmi BR, Dechamma HJ, et al. Cationic microparticle [poly(D,L-lactide-co-glycolide)]-coated DNA vaccination induces a long-term immune response against foot and mouth disease in Guinea pigs. J Gene Med. 2012;14(5):348–352. 22438260 10.1002/jgm.2622

[38] Soliman SM, Sheta NM, Ibrahim B, et al. Novel intranasal drug delivery: geraniol charged polymeric mixed micelles for targeting cerebral insult as a result of Ischaemia/Reperfusion. Pharmaceutics. 2020;12(1):76. 10.3390/pharmaceutics12010076 PMC7022886 31963479

[39] Galluzzi L, Vitale I, Aaronson SA, et al. Molecular mechanisms of cell death: recommendations of the nomenclature committee on cell death 2018. Cell Death Differ. 2018;25(3):486–541. 29362479 10.1038/s41418-017-0012-4 PMC5864239

[40] Martinod K, Witsch T, Farley K, et al. Neutrophil elastase-deficient mice form neutrophil extracellular traps in an experimental model of deep vein thrombosis. J Thromb Haemost. 2016;14(3):551–558. 26712312 10.1111/jth.13239 PMC4785059

[41] Frischbutter S. Guidelines for the use of flow cytometry and cell sorting in immunological studies (second edition). 2019. 10.1002/eji.201970107 PMC7350392 31633216

